# Atrial fibrillation in obesity: Weighing up the evidence for catheter ablation

**DOI:** 10.1002/clc.23416

**Published:** 2020-06-29

**Authors:** Saad Javed, Dhiraj Gupta, Gregory Y. H. Lip

**Affiliations:** ^1^ Liverpool Centre for Cardiovascular Science University of Liverpool and Liverpool Heart and Chest Hospital Liverpool UK; ^2^ Division of Cardiovascular Sciences, Faculty of Biology, Medicine and Health University of Manchester, Health Innovation Manchester Network Manchester UK

The alarming rise in the global prevalence of obesity is paralleled by increasing cases of atrial fibrillation (AF) worldwide, with important implications for AF‐related adverse outcomes.^1^, ^2^ Although the precise mechanisms remain under investigation, data from epidemiological, clinical, and experimental studies have demonstrated the cardiometabolic consequences of obesity both in perpetuating and sustaining AF.^3^ Indeed, in one prospective cohort, every 1 kg/m^2^ increase in body mass index (BMI) conferred a 5% increase in the risk of incident AF.^4^ Bodyweight and BMI fluctuation are also important predictors of incident AF, especially in individuals with low bodyweight and regardless of weight gain or loss.^5^, ^6^ In addition, the management of AF in obesity represents a therapeutic challenge due to a higher burden of comorbidities and changes in pharmacodynamics and pharmacokinetics of various drugs.^7^ Despite these considerations, analysis of data from several studies has reported a paradoxically lower mortality in obese and overweight subjects compared to their leaner counterparts, as well as a lower risk for stroke and major bleeding.^8^ However, whether this obesity paradox is an actual biological effect or not is as yet unclear.

Radiofrequency ablation (RFA) is now an established therapeutic approach for the AF patient with a high symptomatic burden or with disease refractory to drug therapy.^9^ Given the obesity paradox for AF and the plethoric physiological derangements in obesity, the question arises: is RFA in obese subjects associated with poorer outcomes and greater burden of complications? Individual studies have found conflicting evidence in answer to this question.

In this issue of *Clinical Cardiology*, Liu et al attempt to lend greater clarity to this topic in their meta‐analysis examining the impact of BMI for patients with AF undergoing RFA. The authors have pooled data from 10 studies comprising 14 735 obese subjects with AF undergoing RFA to explore the impact of BMI on (a) procedural duration and radiation exposure and (b) outcomes and complications after RFA. From their analysis, several important points emerge. First, as previously reported in literature, the procedure duration and amount of radiation exposure were significantly greater in both obese (BMI ≥30 kg/m^2^) and overweight (BMI ≥25 to <30 kg/m^2^) patients compared to patients with normal BMI.^10^ Second, the risk of specific procedural complications—namely, transient ischemic attack or stroke (overweight, RR: 0.92; obesity, RR: 1.02); cardiac tamponade (overweight, RR: 0.92; obesity, RR 1.02); groin hematoma (overweight, RR: 0.62; obesity, RR: 0.40); and pulmonary vein stenosis (overweight, RR: 0.49; obesity, RR: 0.40)—remained similar across the three considered BMI groups. Third, analyzing the underlying studies reveals that few studies attempt to distinguish between classes of obesity. Indeed, of the 10 studies considered here by Liu et al, only 3 distinguished between classes of obesity. This is particularly important as morbid obesity (ie, BMI ≥40 kg/m^2^) is associated with poorer outcomes following both cardiac surgery and percutaneous coronary intervention.^11^

Shoemaker et al reported in their prospective study that BMI ≥40 kg/m^2^ was associated with a greater risk of complications following AF ablation.^12^ Similarly, Winkle et al observed a significantly higher risk of complications in patients with BMI ≥40 kg/m^2^, although this effect was largely due to minor complications.^13^ These findings, along with the heterogenous nature of the studies considered, seem to suggest that generalizing complication risk for all patients who are overweight or obese is difficult, and indeed, whether an exact threshold for elevated risk exists requires further investigation. It is notable that NHS England has recently released a draft of its proposed commissioning guidelines, according to which AF ablation will not be offered to patients with BMI greater than 40, and patients with BMI between 35 and 40 will need to demonstrate weight reduction of at least 10% of their bodyweight before being eligible for AF ablation (NHS England circular January 7, 2020).

Most studies appear to use BMI as the de facto anthropometric measure of obesity. While BMI is easily calculated, other measures, including waist‐to‐hip ratio or waist circumference, may better capture the degree of adiposity and give a more accurate indication of risk.^14^ Notably, an analysis of the data from one study found that lean body mass was the major risk factor for AF, and other anthropometric measures did not show any correlation with AF after adjusting for lean body mass.^14^ Furthermore, there has been increasing interest in ethnicity‐specific definitions of obesity, and limited evidence suggests that some ethnic groups, particularly Asians, may have higher levels of disease at lower BMI.^15^, ^16^

From a more practical perspective, given these findings by Liu et al, a clinician may ask whether we should advocate weight loss to the obese patient with AF referred for ablation. A number of studies (Table 1) indicate that, in those with established AF, weight loss is associated with a reduction in AF severity and burden, improving both symptoms and recurrence‐free survival.^17^, ^18^, ^19^, ^20^, ^21^, ^22^, ^23^ In the REVERSE‐AF study, every unit decrease in BMI was associated with reduction in progression from paroxysmal to persistent AF and in regression from persistent to paroxysmal AF.^20^ Structurally, weight loss has been associated with reduced left atrial volumes and left ventricular wall thickness.^24^

**TABLE 1:** Selected studies investigating the relationship between weight loss and risk factor management in the context of AF in obesity^17^, ^18^, ^19^, ^20^, ^21^, ^22^, ^23^

Study	Design	Population	Key findings
Abed et al^17^	Randomized controlled	248	Patients with prevalent AF were randomized to weight management (intervention) or general lifestyle advice (control) structured weight management program was associated with significant reduction in AF symptom burden and lower risk of AF recurrence
Alonso et al^18^	Randomized controlled	5067	Patients with type 2 diabetes randomized to intensive lifestyle intervention group or standard diabetes education group Modest weight loss in intervention group Intervention did not affect AF incidence (multivariable HR 0.99, 95% CI 0.77‐1.28) during mean follow up of 9 y
Jamaly et al^19^	Prospective cohort	4021	AF was 29% lower in patients undergoing bariatric surgery vs the control group (HR: 0.71; 95% confidence interval: 0.60‐0.83; *P* < .001)
Middeldrop et al^20^	Observational cohort	355	3% of patients with sustained weight loss ≥10% progressed from paroxysmal to persistent AF compared with 32% who lost 3% to 9% and 48% who lost <3% (*P* < .001). For every one‐unit decline in BMI, there was a 54% reduction in progression from paroxysmal to persistent AF (odds ratio 0.46, 95% CI 0.35‐0.59) and a 71% increase in regression from persistent to paroxysmal AF (OR 1.71, 95% CI 1.41‐2.07)
Pathak et al^21^	Observational cohort	149	Postablation patients assigned to risk factor management o control group. AF frequency, duration, symptoms, and symptom severity decreased more in the RFM group compared with the control group (all *P* < .001)
Pathak et al^23^	Observational cohort	355	AF patients with significant intentional weight loss over a 5‐y follow up (>10%) had sixfold higher likelihood of arrhythmia‐free survival compared with those with modest‐ to no‐weight change (<3%)

Abbreviations: AF, atrial fibrillation; BMI, body mass index; CI, confidence interval; HR, hazard ratio; OR, odds ratio.

Second, while Liu et al did not consider long‐term outcomes following RFA for AF in obese subjects given limitations across different studies, some isolated studies have examined longer‐term postablation outcome data for obese patients with AF. For example, Winkle et al followed patients for 5 years after ablation^24^ and found that BMI ≥35 kg/m^2^ was independently associated with AF recurrence (hazard ratio 1.23; 95% confidence interval 1.02‐1.49). Indeed, lower rates of freedom from AF were found for BMI of 35 to 40 and ≥40 kg/m^2^ groups compared with BMI <35 kg/m^2^. Furthermore, BMI is a dynamic measure, and whether variations in BMI impact postablation outcomes has not yet been evaluated.

**FIGURE 1:**
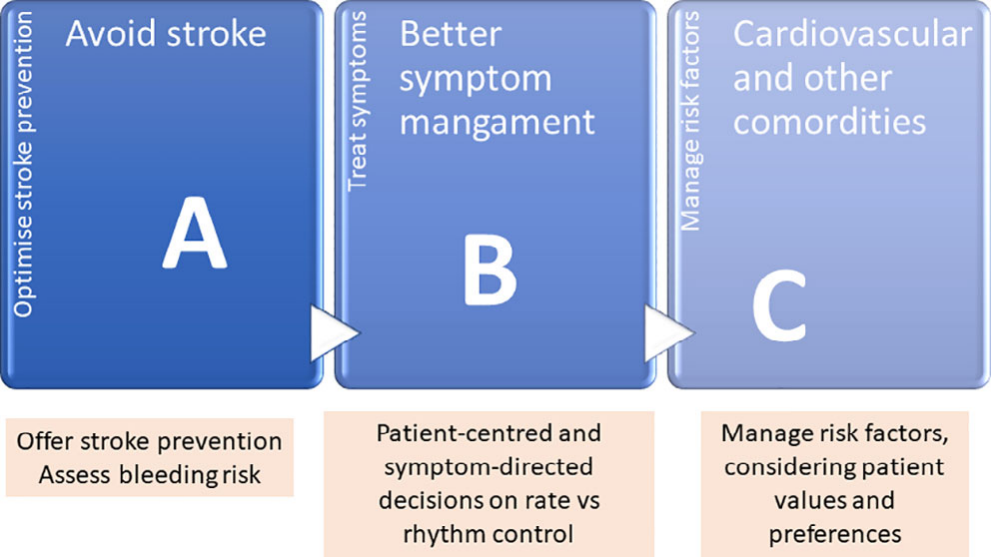
The ABC approach

Taken together, these studies make a compelling argument in favor of a comprehensive risk factor management approach to AF that includes weight loss.^13^, ^20^, ^24^ The “ABC” approach (Figure 1) is one such proposed model that endeavors to unify the conventional “three pillars” of AF management—avoiding stroke/anticoagulation, better symptom management with rhythm or rate control—and cardiovascular/comorbid risk factor management toward a more holistic approach to caring for the patient with AF.^25^

In a similar vein, the present meta‐analysis by Liu et al is an attempt to amalgamate and critically examine the findings from earlier studies evaluating the impact of BMI for AF patients undergoing RFA. The researchers' findings highlight the elaborate nature of the AF‐obesity relationship in the context of ablation, reflecting earlier attempts at decoding this link. Ultimately, this study underscores the need for further research to examine the complex relationship between obesity, weight reduction, and AF in order to add weight to the fourth pillar of AF care: risk factor management.
